# Acceptance of Mobile Health Apps for Disease Management Among People With Multiple Sclerosis: Web-Based Survey Study

**DOI:** 10.2196/11977

**Published:** 2018-12-12

**Authors:** Jennifer Apolinário-Hagen, Mireille Menzel, Severin Hennemann, Christel Salewski

**Affiliations:** 1 Department of Health Psychology Faculty of Psychology University of Hagen Hagen Germany; 2 Department of Clinical Psychology, Psychotherapy and Experimental Psychopathology Institute of Psychology University of Mainz Mainz Germany

**Keywords:** multiple sclerosis, eHealth, mHealth, acceptability of health care, patient preference

## Abstract

**Background:**

Mobile health (mHealth) apps might have the potential to promote self-management of people with multiple sclerosis (MS) in everyday life. However, the uptake of MS apps remains poor, and little is known about the facilitators and barriers for their efficient utilization, such as technology acceptance.

**Objective:**

The aim of this study was to examine the acceptance of mHealth apps for disease management in the sense of behavioral intentions to use and explore determinants of utilization among people with MS based on the Unified Theory of Acceptance and Use of Technology (UTAUT).

**Methods:**

Participants for this Web-based cross-sectional study were recruited throughout Germany with the support of regional MS associations and self-help groups. To identify determinants of intention to use MS apps, a measure based on the UTAUT was adapted with 4 key determinants (performance expectancy, effort expectancy, social influence, and facilitating conditions) and extended by Intolerance of Uncertainty (IU) and electronic health literacy. Potential influencing effects of both MS and computer self-efficacy (C-SE) as mediators and fatigue as a moderator were analyzed using Hayes’s PROCESS macro (SPSS version 3.0) for IBM SPSS version 24.0.

**Results:**

A total of 98 participants (mean age 47.03 years, SD 10.17; 66/98, 67% female) with moderate fatigue levels completed the survey. Although most participants (91/98, 92%) were daily smartphone users, almost two-thirds (62/98, 63%) reported no experience with MS apps. Overall, the acceptance was moderate on average (mean 3.11, SD 1.31, minimum=1 and maximum=5), with lower scores among persons with no experience (*P*=.04) and higher scores among current users (*P*<.001). In multiple regression analysis (*R*^2^=63% variance explained), performance expectancy (beta=.41) and social influence (beta=.33) were identified as significant predictors of acceptance (all *P*<.001). C-SE was confirmed as a partial mediator in the relationship between IU and acceptance (indirect effect: B=−.095, 95% CI −0.227 to −0.01). Furthermore, a moderated mediation by C-SE was shown in the relationship between IU and behavioral intentions to use MS apps for low (95% CI −0.42 to −0.01) and moderate levels (95% CI −0.27 to −0.01) of fatigue.

**Conclusions:**

Overall, this exploratory pilot study indicates for the first time that positive expectations about the helpfulness for self-management purposes and social support might be important factors to be considered for improving the acceptance of MS apps among smartphone users with MS. However, given some inconsistent findings, especially regarding the role of effort expectancy and IU and self-efficacy, the conceptual model needs replication with a larger sample of people with MS, varying more in fatigue levels, and a longitudinal assessment of the actual usage of MS apps predicted by acceptance in the sense of behavioral intentions to use.

## Introduction

### Multiple Sclerosis: Challenges for Self-Management

Multiple sclerosis (MS) is a chronic autoimmune disease of the central nervous system (CNS), which is characterized by exacerbations of neurological dysfunction [1]. Approximately 2.3 million people live with MS worldwide [2], of which half of those affected live in Europe [3] and about 200,000 people live in Germany [4]. Prevalence rates vary between region and registry. The onset of MS is usually between the 20th and 40th year of life, with women being affected more often than men at a ratio between 2:1 [5,6] and 3:1 [3]. Thus, MS is one of the most prevalent neurological disorders in young adulthood, leading to permanent disability and early retirement [3,4].

Principally, 4 major MS forms related to different challenges can be distinguished [6]: with 85% of cases, the most common MS form is relapsing-remitting MS, which is characterized by relapses and exacerbations as well as phases of remission. This form can transit to the secondary progressive form with continuing worsening. The primary progressive form of MS with gradual, continuous worsening from the onset affects approximately 10% of people with MS, whereas progressive relapsing MS with symptom progression from the onset without periods of remission represents less than 5% of cases [6]. Depending on the neuroanatomical localization of plaques of demyelination in the CNS, symptoms can be manifested as various motor, visual, cognitive, and sensory disturbances as well as fatigue [5], making it difficult to predict the individual MS course [6]. Therefore, coping with uncertainties is a key challenge of living with MS [7].

Because little is known about the etiology of MS [5] and there is currently no cure, the long-term MS treatment also has to focus on disease management or self-management and coping with uncertainties [7]. Self-management of a chronic illness can be defined as a dynamic process of actively coping [8]. Given that research has shown that people with MS prefer to take an active role in treatment decisions among most people with MS [9], self-help tools such as mobile health (mHealth) apps for MS could be useful for supporting disease management [10].

### Multiple Sclerosis Apps as Self-Management Tools

Given that people with MS often experience issues in accessing health care services because of barriers such as mobility restrictions, mHealth and electronic health (eHealth) services represent a promising way to facilitate MS disease management [11]. In fact, the internet is the first source for health information for many people with MS [12].

Research suggests that modern technologies and new media in the health context (eHealth) may be helpful for people with MS by promoting adherence, self-management skills [10], mental health [13], physical activity [14-17], and fatigue management [18,19].

Many MS patients in Germany are familiar with using mobile phones for communication with health care providers [20]. Furthermore, a recent survey of the North American Research Committee on Multiple Sclerosis registry showed that 28.6% of participants with MS have used secure Web-based portals for the exchange of medical information with health care providers and that 46.2% of the smartphone and tablet users used an mHealth app [21].

Moreover, mHealth apps appear especially suitable for people with MS because of their location-independent and time-flexible accessibility [11]. There is also a growing number of positive economic evaluations on the cost-effectiveness of mHealth apps for medical conditions [22]. Hence, high-quality mHealth apps for MS can further help empower people with MS to be more active in their disease management and informed decision making [23].

The focus of existing apps and other stand-alone or blended care eHealth solutions lies in screening and assessment, disease monitoring and self-management, advice and education, as well as treatment and rehabilitation [11]. A narrative review of 28 eHealth and mHealth solutions for MS by Marziniak et al [11] showed that mHealth apps for MS patients such as Msdialog, COGNI-TRACK, or MyBetaApp usually address self-management and monitoring (eg, medication reminder and symptom tracking), whereas Web-based interventions such as Deprexis or MS Invigor8 focus on treatment and rehabilitation [11].

However, despite these advantages, the uptake of MS apps is poor. A scoping review [24] indicated that most MS apps for disease management failed to meet the needs and demands of users with MS. While advantages of eHealth and mHealth solutions include the possibility to connect with others [25], improved health care access or greater independence [26], perceived disadvantages involve the potentially poor usability for people with neurological impairments [25], and data security concerns [26]. However, especially in countries such as Germany with an early stage of eHealth adoption in routine care [27], little is known about the acceptance of mHealth services for MS as another barrier.

To shed light on the determinants of MS apps’ uptake, technology acceptance models (TAM) [28] such as the Unified Theory of Acceptance and Use of Technology (UTAUT) [29] provide guidance as a validated framework. In these models, acceptance is operationalized as behavioral intention to use or actual usage of a technological innovation as dependent variables of a set of personal and interpersonal attitudinal factors. In the context of the TAM, Davis et al [28] argued that people form attitudes and intentions toward learning to use a novel technology, which are associated with uncertainties, before starting efforts aimed at performing. As an early form of acceptance, intention to use represents a well-established predictor of behavior, for instance, in terms of health behavior [30] and the use of psychological services [31] as well as information technology [28]. In accordance with the Theory of Planned Behavior (TPB [30]), behavioral intention can be understood as an evaluation or attitude toward a behavior, affecting the likelihood of performing a particular behavior.

The UTAUT [29], which was evaluated in organizations in which learning to use a novel technology was either voluntary or mandatory for employees, is a synthesis of 8 validated models such as the TPB [30]; TAM [28,32]; the Diffusion of Innovation theory [33]; and the Social Cognitive Theory [34], hypothesizing a total of 32 constructs and up to 4 moderators (ie, gender, age, voluntariness, and experience) [29]. According to the UTAUT [29], performance expectancy (eg, perceived usefulness), effort expectancy (eg, ease of use), and social influence (eg, subjective norm) are predictors of the intention to use a technology, whereas facilitating conditions (eg, support and compatibility) and behavioral intention are direct determinants of usage behavior in business organizations.

In recent years, the UTAUT and TAM have been implemented in various medical settings [35-41]. However, to the knowledge of the authors, no study to date has modified the UTAUT model to the acceptance of MS apps.

On the basis of the theoretical considerations and empirical findings from other medical contexts [35], it can be hypothesized that the intention to use MS apps, as an early form of acceptance, is higher, in case of high degrees of the perceived usefulness of MS apps for self-management (*performance expectancy*), the expected ease of use (*effort expectancy*), the approval as being helpful by significant others (*social influence*), and available facilitating factors related to the use of MS apps such as technical support (*facilitating conditions*).

For the specific context of MS apps, further MS-related and technology-related variables could be relevant to understand their acceptance. For instance, research indicates that Intolerance of Uncertainty (IU) with respect to problem-focused coping [7] and eHealth literacy [42] might be additional predictors of health behavior and disease management in MS.

Moreover, self-efficacy, defined as the personal belief in one’s capability to overcome challenges with respect to MS (multiple sclerosis self-efficacy, MS-SE [43]) and using technology (computer self-efficacy, C-SE [44]), might influence the acceptance of MS apps.

Fatigue is a common disabling condition in MS [45], with about three-thirds of people being affected by severe fatigue (compared to NARCOMS, 74% [46]). Hence, fatigue might play a moderating role in behavioral intention to use MS apps.

### Goals of This Study

The purpose of this pilot study was to explore factors influencing the acceptance of MS apps among smartphone users with MS in Germany. We hypothesize that the expectations and beliefs associated with the use of MS apps, IU, and eHealth literacy will significantly predict the acceptance of MS apps. We assumed a significant predictive contribution of the following core UTAUT determinants in the behavioral intention to use MS apps (in the sense of early acceptance): (a) performance expectancy, (b) effort expectancy, (c) social influence, (d) facilitating conditions as well as extended predictors, (e) IU, and (f) eHealth literacy. We expected significant positive associations between the predictors of the UTAUT determinants and eHealth literacy behavioral intention MS apps and a significant negative association between IU and behavioral intention MS apps. Our research questions are as follows: Does self-efficacy explain and fatigue influence the hypothesized relationships between the determinants proposed under hypothesis 1 and acceptance of MS apps? Consequently, another goal was to determine mediating effects of (a) MS-SE and (b) C-SE (research question 1a and b), and moderating effects of fatigue in the relationship between the 6 predictors and behavioral intentions to use MS apps (research question 2).

## Methods

### Study Design and Setting

We conducted a Web-based cross-sectional survey. Data were collected anonymously between March 8, 2017, and April 15, 2017, using Unipark (Enterprise Feedback Suite survey, version Spring 2017, Questback). No ethical approval was required by the institution of the principal investigators because it was an anonymously conducted, self-selected, and voluntary Web-based survey study that involved no intervention, no deception, and no potentially adverse or burdensome questions or tests. Participants were required to give informed consent to participate in the study using Unipark (click-to-agree). No monetary compensation was offered for participation. The average completion time was 15 min.

### Participants and Recruitment

In this Web-based pilot study using convenience sampling, we were interested in the opinions of smartphone users with MS. As this study aimed to include only people with diagnosed MS over the age of 18 years, participants were recruited via a letter to the national associations of the German Multiple Sclerosis Society (“Deutsche Multiple Sklerose Gesellschaft” [DMSG]). Overall, 11 out of 16 regional associations accepted the invitation to share the link to the study via their websites, Facebook profiles, and newsletters. In the 5 other federal states, the recruitment took place by inviting 25 local MS self-help groups via email. In addition, there was a call via the online platform “MSlife” (Biogen, Germany).

A priori power analyses using G*Power, version 3.1 [47], (*F* tests, multiple regression: Omnibus, *R*^*2*^ deviation from 0) yielded a required sample size of at least 77 participants to determine a moderate-to-high effect of f^2^=0.3 (alpha=.05; power=0.95) for the multiple regression model with 6 predictors (critical *F*_6,70_=2.23, noncentrality parameter λ=23.10). The power of 0.95 was chosen based on the assumption of low risk of false negatives with this study design. The effect size was chosen based on a previous work on the UTAUT [48] and a study using a UTAUT-questionnaire design we adapted [35], showing high explained variance. Because there was no study using the same measure, we decided to use the squared correlation *R*^*2*^=.25 to calculate the effect size f^2^. This resulted in the effect size of f^2^=0.33, which we rounded to f^2^=0.3.

### Formulation of a Conceptual Model for the Acceptance of Multiple Sclerosis Apps and Its Operationalization: Adaptation of the Unified Theory of Acceptance and Use of Technology Framework

**Figure 1:**
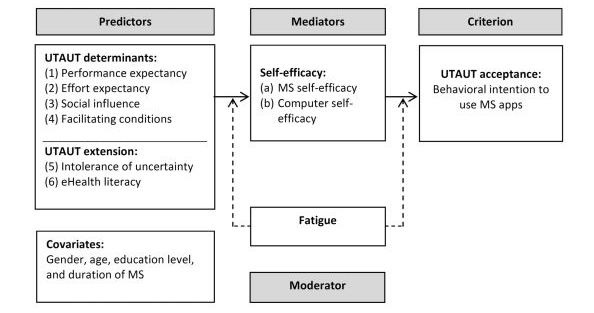
Conceptual study model: adapted and extended Unified Theory of Acceptance and Use of Technology for the acceptance of multiple sclerosis apps. UTAUT: Unified Theory of Acceptance and Use of Technology; MS: multiple sclerosis; eHealth: electronic health.

Because the UTAUT [29] originates in organizational contexts, the constructs were adapted and extended to the context of MS apps (Figure 1). Items from the UTAUT model were adapted to MS apps for smartphones based on the original questionnaire [29] as well as an adapted measure for Web-based aftercare in Germany [35] and extended with the 2 additional predictors IU and eHealth literacy. To minimize the risk of overload because of an excessive number of items, truncated scales were used. For the items we adapted from the English version of scales, forward translation was used, which was checked independently by 3 professionals, of which 2 had a scientific or psychological background (PhD level and BSc level) and the other 1 was an English teacher. Furthermore, various items of prior studies using adaptations of the UTAUT measure were available in German through contacting the study’s authors [35]. The 8-item German eHealth Literacy Scale (G-eHEALS) [49] was also available in German. Testing the transadaption was performed using cognitive debriefing. To ensure the comprehensibility of items adapted from scales available in German or English, 5 students were asked to give their feedback independently from each other. Furthermore, the final Web-based survey was pretested by 13 external persons from the personal network of the second author to avoid technical problems. All items we used to assess the conceptual model are presented in Multimedia Appendix 1.

### Variables and Measures

#### Scales of the Adapted and Extended Unified Theory of Acceptance and Use of Technology Model

The overall survey consisted of 60 items, of which 40 items were used for the assessment of the model (16 items for UTAUT variables, 20 items for additional variables, and 4 items for the covariates).

In all adapted scales with numerical variables, participants were asked to indicate their agreement to statements on a 5-point Likert scale ranging from 1 (“fully disagree”) to 5 (“fully agree”).

#### Acceptance: Behavioral Intention to Use Multiple Sclerosis Apps as Criterion

As an indicator of early acceptance, behavioral intention was operationalized as the plan to use MS apps for disease management purposes. Behavioral intentions to use MS apps in general and within the next 4 weeks were measured using 3 items from the original UTAUT [29] we adapted. The sample was divided into 3 groups who received slightly modified items, considering actual use of MS apps in relation to intentions to use: participants who currently use MS apps (group 1=users), participants who have never used MS apps before (group 2=nonusers), and those who used MS apps in the past (group 3=past users). We asked current users if they would also use MS apps in the future, whereas past users were asked if they intend to use MS apps again. Nonusers received similar but more generally formulated items (Multimedia Appendix 1). A total scale score unifying the responses of the 3 groups was generated for the regression analysis, in which the mean value of the 3 items targeting acceptance for each participant was calculated and transferred to the total scale.

#### Predictors of Acceptance of the Extended Unified Theory of Acceptance and Use of Technology Model

##### Unified Theory of Acceptance and Use of Technology: Performance Expectancy

Performance expectancy is defined as the extent to which a person believes that using a technology could improve outcomes and has the strongest predictive value for acceptance in the UTAUT [29]. In this study, performance expectancy was operationalized as the expectation of a person with MS that using MS apps would be helpful for disease management purposes.

Performance expectancy was assessed with 4 items used by Hennemann et al [35], which we adapted to assess expected outcomes in connection to the perceived usefulness or helpfulness of MS apps. Because social participation is essential for successful adjustment to MS [50], a fifth item was added to the survey (“Using MS apps could help me maintain social contacts.”).

##### Unified Theory of Acceptance and Use of Technology: Effort Expectancy

Effort expectancy is one’s belief about how easy it is to use a technology, taking its complexity and difficulty into account [29]. In this study, effort expectancy is defined as the extent of perceived ease with which MS apps can be used.

Effort expectancy was evaluated with 2 adapted items based on studies in inpatient medical settings and the original UTAUT [29,35] (eg, “I suspect that using MS apps would be easy”).

##### Unified Theory of Acceptance and Use of Technology: Social Influence

Social influence refers to the extent to which a person believes that relevant others think one should perform the behavior in question and that the technical innovation could be useful in relation to a particular goal [29]. In this study, social influence in the sense of subjective norm was measured by asking participants to assess the extent to which (1) their close family members, (2) primary care provider, and (3) friends would consider the use of MS apps helpful for disease management. An *other* response option was provided so that respondents could add examples of other groups of people, but no additional groups were added.

##### Unified Theory of Acceptance and Use of Technology: Facilitating Conditions

Facilitating conditions are defined as a person’s belief that organizational and technical support is available when using a novel technology. This construct also involves the extent to which an innovation is experienced as compatible with personal values, needs, and experiences; perceived behavioral control; and objective factors [29]. Among people with MS, the plan to using a new technology despite a disabling long-term condition might be affected by perceived available resources, such as knowledge and support options.

Facilitating conditions were assessed based on 3 items, of which 2 items were completely adopted and adapted from a study in inpatient medical settings [35]. The third additional item was created based on the original UTAUT [29], which considers social support as a facilitating condition (“If I had problems using MS apps, I would know where to get help.”).

##### Intolerance of Uncertainty

People with MS are confronted with numerous disease-related uncertainties [51]. High levels of IU are associated with increasing efforts to regain control of the uncontrollable situation, which can result in dysfunctional coping strategies [7]. IU is often related to incapacitation, stress-inducing perceptions, and a tendency to avoidance [52]. Under the assumption of using MS apps as a strategy for problem-focused coping with respect to disease management strategies, IU could have a negative influence on intentions to use.

Of the 27-item IU Scale (IUS [52]), this study used 4 items with the highest factor loadings per subscale from the primary study using a student sample (items 5, 12, 19, and 16). In total, 3 items were only translated, for instance, “My mind can’t be relaxed if I don’t know what will happen tomorrow” (item 12 of the IUS). We only adapted 1 item by adding a relationship to a *disease* (“When it’s time to act, uncertainty associated with my disease rather paralyzes me”).

##### Electronic Health Literacy

To be able to take an active role in medical decision making and self-management, people with MS require adequate information [51], which they often seek online [20]. eHealth literacy is defined as the ability to search, find, understand, evaluate, and use health information available via electronic resources [53]. As eHealth and mHealth tools for MS can improve self-management skills [10], it can be assumed that higher eHealth literacy may increase the likelihood of effectively using electronic resources such as MS apps [54].

eHealth literacy was measured using 4 slightly modified items of the 8-item G-eHEALS [49]. From the subscale *information search* (6 items), 2 items with the highest factor loadings were selected, whereas both items comprising the *information evaluation* were included.

### Mediator Effects of Self-Efficacy Beliefs

#### Multiple Sclerosis Self-Efficacy

MS-SE can be understood as one’s confidence in the ability to handle challenges related to MS [43]. Self-efficacy has been shown to predict health-related behavior in people with MS, including physical mobility [55-57], psychosocial adjustment [43], or pain-related coping strategies [58], and to mediate health-related relationships [59,60]. Thus, it can be assumed that MS-SE can help explain problem-focused coping strategies such as using MS apps.

MS-SE was assessed using the 11-item Liverpool Self-Efficacy Scale [61]. For economic reasons, 2 items per subscale (control and personal agency) were chosen based on the criterion of face validity. We added the term *MS* in the German translation, for instance, “Despite my difficulties, I still manage to cope with daily life with MS.”

#### Computer Self-Efficacy

C-SE is defined as the personal belief regarding one’s ability to use computer technology to perform specific tasks [62]. A meta-analysis of 102 studies [44] confirmed that C-SE is associated with behavioral intention and usage behavior as well as with components of technology acceptance such as perceived usefulness and ease of use. Therefore, we will examine the extent to which C-SE with respect to MS apps can explain the proposed relationships.

A measure of general computer self-efficacy (GCSE [63]) was used, but *computer* was replaced by *smartphone*. For economic reasons, the original 5-item scale was limited to the 3 items with the highest factor loadings (items 2, 3, and 5), for instance, “I believe I have the ability to remove apps from my smartphone I no longer need” (adapted item 5 of the GCSE).

### Moderator Effects of Fatigue

Fatigue can be described as a state of subjective physical or mental exhaustion, varying largely in intensity over the day course [64,65]. Such fluctuations can hinder simple routines, job performance, and social activities, making fatigue one of the main reasons for incapacity to work [64,66]. On the one hand, MS apps such as *MoreStamina* [67] could be used on a compensatory basis for the management of fatigue. On the other hand, fatigue might also be one reason for the poor uptake of MS apps, for instance, because of negative effort expectancies. Hence, a moderating role of fatigue appears possible.

For the retrospective assessment of fatigue in the past 4 weeks, the 5-item Modified Fatigue Impact Scale [68] was used.

#### Control Variables

In the original UTAUT, age, gender, voluntariness (of learning to use a technology vs mandatory use in organizations), and experience were confirmed as moderators for the key relationships [29]. To control their influence in this study, age and gender were included as covariates in the mediation and moderation models. Gender as a categorical variable was included with dummy coding (0=male and 1=female). Age and duration of MS were included as numerical variables. Because using MS apps as a self-help tool is a voluntary choice, voluntariness was no applicable variable in this study. Experience was operationalized as the duration of MS, not as experience with MS apps. Due to the unclear proportion of MS app users in the target population, we found that the duration of living with MS might be a more meaningful indicator for experience with disease management. Furthermore, the education level was considered, as studies indicate a more likely use of eHealth services among higher educated people with MS [69]. An MS Registry survey [21] found a higher likelihood of smartphone, tablet, and mHealth app use being associated with younger age and higher education in people with MS. In line with the Comparative Analysis of Social Mobility in Industrial Nations (CASMIN-classification [70]), the educational attainment was assessed as an ordinal scale in 3 levels ranging from 1 (“low”) to 3 (“high”).

### Procedure and Scale Metrics

The Web-based survey comprised a total of 60 items and optional commentary fields. The first part of the survey consisted of sociodemographic (6 items) and MS-related (4 items) questions using nominal scales.

Then, the MS-related constructs fatigue (5 items), IU (4 items), and MS-SE (4 items) were assessed on 5-point Likert scales. Smartphone use and frequency of use (2 items) as well as usage preferences (4 items) were asked using nominal scales. eHealth literacy (4 items) and C-SE (3 items) were evaluated on 5-point Likert scales. Use of MS apps (5 items) was assessed on a nominal scale. Overall, 3 optional questions (free text fields) were asked about subjective benefits, challenges, and suggestions for improvements regarding MS apps. To summarize the responses, the UTAUT model was used to map the responses to categories following the approach of a quantitative content analysis. Finally, 16 items on a 5-point Likert scale were used to measure UTAUT variables: behavioral intention to use (3 items), performance expectancy (5 items), effort expectancy (2 items), social influence (3 items), and facilitating conditions (3 items).

### Statistical Analysis

Only completed surveys were considered for data analyses (listwise deletion). No imputation technique was used to compensate missing values because the vast majority of dropouts occurred after the first 3 demographic questions (missing not at random). Descriptive analyses were performed to obtain information on sociodemographics and usage of modern technology for MS-related purposes. Both simple and multiple linear regression analyses were conducted to determine predictors for the acceptance of MS apps. Due to the exploratory nature of this study and as the limited evidence base related to the proposed relationships in this specific model was too scarce, the predictors were included simultaneously in the multiple regression model. All analyses were performed using SPSS, version 24 (IBM Analytics), in which the macro PROCESS by Hayes [71] was implemented to test mediation (C-SE and MS-SE) and moderation hypotheses (fatigue). Before the analyses, the assumptions of multiple linear regression analysis were confirmed being sufficiently fulfilled to perform parametric tests. Data analyses were performed independently and cross-checked by 2 researchers. The significance level for all hypotheses was alpha <.05.

## Results

### Descriptive Analyses

The survey platform was accessed 496 times. In total, 175 people agreed to participate (informed consent), of which 113 people fully completed the survey (attrition rate of 35.4%). Most participants (52/62, 84%) who dropped out of the survey did so after the first 3 demographic questions (ie, year of birth, gender, and postal code). The data of the other 15 participants were eliminated because of incomplete data because they indicated they did not possess a smartphone. Because the target sample was smartphone users, data from these participants were not included in the analysis.

#### Sample Characteristics

As shown in Table 1, the final sample consisted of 98 people aged between 22 and 67 years (median 48.0 years, interquartile range 14.0 years). The CASMIN-based mean score (mean 2.23, SD 0.61) indicated a moderately high education level [70]. Detailed sample characteristics are presented in Multimedia Appendix 2.

#### Frequency and Purposes of Mobile Phone Use

**Figure 2:**
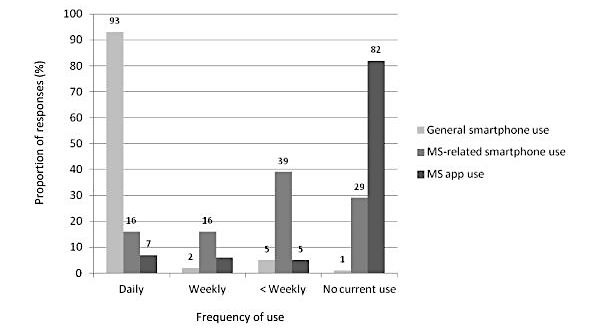
Frequency of general and multiple sclerosis–related smartphone use, proportions in percent (N=98). MS: multiple sclerosis.

Figure 2 shows the frequency of using the mobile phone or smartphone in general and for MS-related purposes.

In terms of MS-specific use, the mobile phone or smartphone was mostly used as internet access to search MS information or connect with others (51/98, 52%), scheduling (eg, for medical consultations, 45/98, 46%), making calls for medical purposes (44/98, 45%), MS-related emails or text messages (34/98, 35%), and for music or games (eg, for relaxation and cognitive training, 19/98, 19%). Other use (8/98, 8%) included apps as medication reminders. In total, 19% (19/98) did not answer this question.

**Table 1:** Sample characteristics (N=98).

Variables		Statistics
**Age (years)**		
	All, mean (SD), range	47.03 (10.17), 22-67
	Women, mean (SD), range	45.11 (10.09), 22-67
	Men, mean (SD), range	51.0 (9.28), 22-66
	20-35 years, n (%)	16 (16)
	36-50 years, n (%)	43 (44)
	51-67 years, n (%)	39 (40)
**Gender, n (%)**		
	Female	66 (67)
	Male	32 (33)
**Secondary education, n (%)**		
	Certificate of secondary education^a^	8 (8)
	General certificate of secondary education^b^	27 (28)
	Advanced technical college entrance qualification^c^	19 (19)
	General qualification for university entrance^d^	44 (45)
**Vocational training and tertiary education, n (%)**		
	No professional qualification	4 (4)
	Training qualification^e^	62 (63)
	Polytechnic or college degree	9 (9)
	University degree	23 (24)
**Duration of multiple sclerosis (years)**		
	All, mean (SD), range	13.92 (9.84), 1-45
	1-10, n (%)	43 (44)
	11-21, n (%)	37 (38)
	>21, n (%)	18 (18)

^a^German “Hauptschulabschluss” as basic school qualification.

^b^German secondary school level-I certificate (“Mittlere Reife”).

^c^German “Fachhochschulreife.”

^d^German “Allgemeine Hochschulreife” (“Abitur” or A Level).

^e^German dual training model.

#### Use of Multiple Sclerosis Apps for Disease Management Purposes

The majority of participants (62/98, 63%) reported no experience with MS apps. Of the 36 participants (36/98, 37%) reporting experience with MS apps, 18 people (18/36, 50%) were currently using them.

Most of the 36 participants with experience with MS apps indicated the use of the app *MS Kognition* (MS cognition) by the DMSG (15/36, 42%) in the comment field. Further apps reported by the participants are presented in Multimedia Appendix 2. In addition, Multimedia Appendix 2 provides a summary of optional free text responses on perceived benefits and challenges of MS apps as well as ideas for improvements.

Specific MS apps were used for purposes of cognitive training to improve attention or concentration (12/18, 67%), information and education about MS (9/18, 50%), reminders of appointments or medication intake (8/18, 44%), documentation (6/18, 33%), maintaining social contacts (6/18, 33%), and strengthening physical skills (3/18, 17%).

In addition to MS apps, 48% (47/98) participants also used other (non-MS-specific) mHealth apps for disease management, mostly for cognitive training (23/47, 49%); strengthening physical well-being, including yoga and fitness (12/47, 26%); orientation in public life (eg, finding barrier-free places, 10/47, 21%); nutrition (8/47, 17%); stress management (3/47, 6%); mood management (eg, anxiety and depression, 2/47, 4%); and other purposes (7/47, 14.9%). The awareness of the existence of internet-based therapies was low (aware: 25/98, 26%; not aware: 67/98, 68%; and not sure: 6/98, 6%).

#### Descriptive Analysis of the Scales Related to the Acceptance of Multiple Sclerosis Apps

Table 2 summarizes the mean values, SDs, and Cronbach alpha for each numerical scale (N=98). As shown in Table 2, the overall acceptance was moderate (mean 3.11, SD 1.31). When compared with the overall mean score, participants reporting no experience with using MS apps expressed significantly lower acceptance (mean 2.76, SD 1.32; *t*_61_=–2.100; *P*=.04) and current users had significantly higher acceptance scores (mean 4.33, SD 0.79; *t*_17_=6.552; *P*<.001), whereas the difference with former users was not significant (mean 3.11, SD 0.91; *t*_17_=0.005; *P*=.996).

### Principal Results of Regression Analyses

#### Multiple Regression Analysis

Correlation analyses and simple regression analyses (Multimedia Appendix 3) showed significant correlations between the variables and a significant contribution of the variables, except for eHealth literacy, in behavioral intentions to use.

According to the *F* test (*F*_6,91_=25.702), the overall regression model contributes 63% of explained variance (*R*^*2*^=.625; *P*<.001). The additional inclusion of the 4 control variables would have only yielded in a marginally increased explained variance of 1.3% (up to *R*^*2*^=64.3%). As shown in Table 3, with a regression coefficient of B=.63 (beta=.41; *P*<.001), performance expectancy proved to be a significant predictor of intention to use as well as social influence with B=.42 (beta=.33; *P*<.001). Contrary to hypothesized, the other predictors had no meaningful influence on acceptance in the multiple regression model (all *P*>.05).

**Table 2:** Mean values, SDs, and internal consistency of the scales of the conceptual study model (N=98).

Variable or scale^a^		Mean (SD)^b^	Cronbach alpha^c^
Behavioral intention to use overall (3 items per group, N=98)^d^		3.11 (1.31)	—^e^
**Behavioral intention to use per group**			
	Group 1: current users (n=18)	4.33 (0.79)	.83^f^
	Group 2: nonusers (n=62)	2.76 (1.32)	.91^g^
	Group 3: past users (n=18)	3.11 (0.91)	.73^h^
Performance expectancy (5 items)		2.81 (0.97)	.88^f^
Effort expectancy (2 items)		3.80 (0.80)	.60^i^
Social influence (3 items)		2.81 (1.02)	.90^g^
Facilitating conditions (3 items)		4.45 (0.78)	.85^f^
Intolerance of uncertainty (4 items)		2.61 (0.99)	.78^j^
Electronic health literacy (4 items)		4.22 (0.70)	.87^f^
Multiple sclerosis self-efficacy (4 items)		4.06 (0.80)	.85^f^
Computer self-efficacy (3 items)		4.18 (0.94)	.84^f^
Fatigue (5 items)		3.31 (1.17)	.89^f^

^a^Items were adapted from previous research (Multimedia Appendix 1).

^b^Scale range; minimum=1 to maximum=5. Item keying: higher scores mean a higher expression of the respective variable.

^c^Internal consistency; classification according to Cohen criteria [72].

^d^Group 1=participants who are current users of MS apps, group 2=participants who never used MS apps, and group 3=participants who had used MS apps in the past. All assessed 3 items on behavioral intention that were modified based on the experience with MS apps.

^e^Not applicable.

^f^Cronbach alpha: good.

^g^Cronbach alpha: excellent.

^h^Cronbach alpha: sufficient.

^i^Cronbach alpha: questionable.

^j^Cronbach alpha: acceptable.

**Table 3:** Coefficients in the multiple regression model of the adapted and extended Unified Theory of Acceptance and Use of Technology (N=98).

Predictors^a^	B	SE	Beta	*t* test	*P* value	Tolerence	VIF^b^
Constant	−.56	0.77	—^c^	−0.73	.47	—	—
Performance expectancy	.63	0.12	.47	5.32	<.001	.52	1.92
Effort expectancy	.09	0.13	.06	0.70	.49	.62	1.6
Social influence	.42	0.13	.33	3.33	.001	.42	2.40
Facilitating conditions	.07	0.13	.04	0.51	.61	.64	1.56
Intolerance of uncertainty	.09	0.09	.06	0.90	.37	.79	1.27
Electronic health literacy	−.04	0.14	−.02	−.030	.77	.78	1.28

^a^Criterion: behavioral intention to use MS apps. All predictors were included simultaneously, without covariates.

^b^VIF: variance inflation factor.

^c^Not applicable.

**Figure 3:**
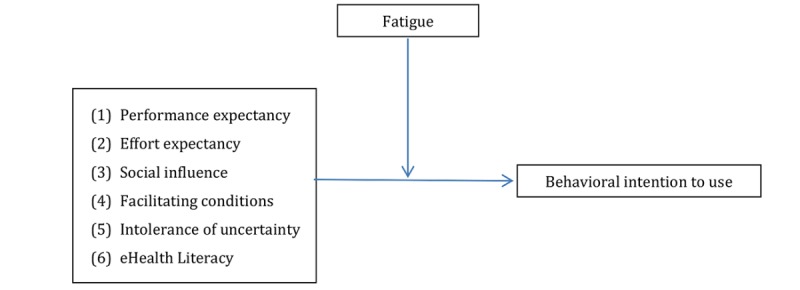
Exploratory model for the assessment of the moderation hypotheses for fatigue. Criterion: behavioral intentions to use multiple sclerosis apps. The numbering of the predictors corresponds to the numbering of the 6 reported models. eHealth: electronic health.

#### Mediator Effects of Self-Efficacy

Mediation hypotheses were examined individually for each predictor (Multimedia Appendix 3). Due to the correlation of both mediators (*r*=.289; *P*=.004), a multiple mediation analysis was performed in which both C-SE and MS-SE were successively tested.

However, the assumption of mediation could only be confirmed for C-SE in the model specification with IU as a predictor of behavioral intention (model 5). Both the effect of IU on C-SE (a path, B=−.23; *P*=.01) and C-SE on behavioral intention (b path, B=.42; *P*=.006) were significant. The significant indirect effect of C-SE (B=−.095, 95% CI −0.227 to −0.01) suggests the reduction of the direct effect (c path, B=.29; *P*=.046) compared with the total effect (c path, B=.30; *P*=.02), indicating a partial mediation.

Furthermore, the control variable age showed significant influence on C-SE (B=−.03; *P*=.003), as did gender (B=−.45; *P*=.02), meaning that younger and male participants had a stronger expression of C-SE.

#### Moderator Effects of Fatigue

Simple regression analysis confirmed fatigue as a significant positive predictor of behavioral intention (*R*^*2*^=.05; B=.24; beta=.21; SE=.11; *P*=.04). As illustrated in Figure 3, each predictor was examined individually for the effect of fatigue.

Contrary to hypothesized, the interaction terms (predictor × moderator) as an indicator for moderation effects of fatigue were not significant for performance expectancy (B=−.01; *P*=.92), effort expectancy (B=.22; *P*=.08), social influence (B=−.07; *P*=.37), facilitating conditions (B=.05; *P*=.73), IU (B=−.06; *P*=.58), and eHealth literacy (B=.09; *P*=.57). Because there was at least a marginal interaction effect (*P*=.08), inferential statistics were applied, which showed a significant fatigue-related effect of effort expectancy on behavioral intention for average (95% CI 0.23 to 0.86) and high levels of fatigue (95% CI 0.33 to 1.28; all *P*<.001), as shown in Multimedia Appendix 3.

As mediation effects of C-SE could only be confirmed in the relationship between IU and intentions to use MS apps, only this model was tested for moderated mediation. For this purpose, the moderated mediation index was calculated for the moderator’s mean score (±1 SD). The results indicated a significant conditional indirect effect for both low (95% CI −0.42 to −0.01) and moderate levels (95% CI −0.27 to −0.01) of fatigue. Hence, the indirect effect of C-SE on the relationship between IU and behavioral intention varies depending on the levels of fatigue.

## Discussion

### Principal Findings and Comparison With Prior Work

The aim of this study was to assess determinants of acceptance of MS apps for disease-related self-management purposes, taking into account possible mediating effects of self-efficacy and a moderating role of fatigue.

### Predictors of the Acceptance of Multiple Sclerosis Apps

Within the multiple regression analysis of the adapted UTAUT model, the explained variance of 63% proved to be relatively high but was lower than in the original UTAUT (70% [29]) and previous work (78% [35]). In line with previous research [29,48], performance expectancy was replicated as the strongest predictor in this model. Furthermore, the significant predictive contribution of social influence and the insignificant relationship between facilitating conditions and behavioral intention were also shown in a study investigating acceptance of medical aftercare in inpatients by Hennemann et al [35].

Considering the significant role of social influence in mHealth acceptance, an extension to digital sources of support could be considered such as social media. People with MS are usually well informed about the disease, but, at the same time, appear being vulnerable to scientifically not proven health information and hopes of cure, especially on social media websites [73]. Although most people with MS use the internet as the first choice for health information, their physician remains the most trusted source [12]. Furthermore, there is preliminary evidence that the spread of misinformation about MS therapy options is lower or less influential in social media networks for laypeople with MS under the presence of medical experts [74] than in open-access not moderated MS forums [75]. Social media appears to be a relevant information source for people with MS, especially in terms of sharing opinions and experience with MS [75]. Hence, the beliefs and attitudes of other people with MS within social networks (bottom-up process) and health care providers (top-down process) should be considered in affecting the acceptance of MS apps.

Contrary to the previous empirical findings [76], effort expectancy proved to be an insignificant predictor in the multiple regression analysis, whereas it was significant as a single predictor. This is contrary to another study by Chua et al [77] that showed the relevant effect of effort expectancy besides social influence and performance expectancy in the acceptance of social media apps. One reason for this could be methodological shortcomings of the 2-item scale, given the questionable internal consistency (alpha=.60), as well as common variance with other constructs. Another reason could be the low experience with the usability of MS apps. In addition, effort expectancy could be less relevant in this sample scoring relatively high on eHealth literacy and C-SE. With reference to Venkatesh et al’s study [29], it can be further assumed that by including actual behavior, a stronger predictive weighting of effort expectancy could have been achieved.

Moreover, IU was not significant in the overall model anymore, which should be critically seen in view of methodological issues with this construct [78] and inconsistent findings regarding its effects on coping with MS [7].

Nonetheless, IU made at least a significant contribution to predicting acceptance in simple regression analysis. However, this relationship was positive and not negative as expected. This unexpected finding suggests that IU seems to be associated not only with incapacitation and avoidance [7,52,79] but may also result in functional problem-focused coping strategies such as increased willingness to use modern self-help tools. However, to identify which factors actually influence whether IU manifests itself through functional or dysfunctional outcomes needs further exploration [7]. It is also important to note that using mHealth apps could be emotion-focused, for instance, for stress management purposes [80]. Potentially, this study had an overly narrowed view of the problem-focused function of MS apps such as cognitive training or medication reminders.

In contrast, perceived eHealth literacy was the only variable without significant predictive value. Potentially, this construct is not suitable for the measurement of mHealth literacy in this specific context, as the construct validity has been debated [81]. Furthermore, the construct seems too restricted to Web-based information and not related to other Web-based self-management activities in long-term conditions. Another potential reason is ceiling effects with respect to the identified high eHealth literacy scores in this self-selected Web-based sample. A further investigation of health literacy in a more diverse population appears reasonable because research indicated that, for instance, functional literacy is associated with higher comfort levels and perceived skills with using eHealth information [42] and that many people with MS are quite willing to using eHealth services [21].

Taken together, the theoretical and empirical validation of an extended UTAUT model for MS apps and related innovative tools can mainly rely on the classical determinants, when they are adapted to the MS context. In contrast, the current evidence base for further constructs appears too limited and inconsistent.

### Mediating and Moderating Effects Involved in the Acceptance of Multiple Sclerosis Apps

Another aim of this study was to investigate the role of self-efficacy as mediator and fatigue as moderator. The findings suggest that the hypothesis of mediation for MS-SE must be rejected in all models. Evidence of partial mediation by C-SE was found in the case of IU predicting intentions to use MS apps. An explanation for this finding might be found in the social cognitive theory (SCT [34]). SCT proposes that the concept of self-efficacy is based primarily on the conviction that one is actually able to perform a certain behavior. It may be possible that the influence of self-efficacy has been mitigated by the decision to include only the intention to perform a behavior as a criterion in the model. William and Rhodes [82] argued that the self-efficacy construct is confounded and represents the motivation rather than one’s perceived capability to perform a health-related behavior.

Although fatigue represents a common disabling symptom in MS [19], no moderation effect was identified. Only a marginally significant relationship between effort expectancy and behavioral intention was found, indicating that the expected ease of use related to using MS apps could be higher under the influence of average and higher levels of fatigue. In comparison with clinical samples, the moderate fatigue levels in the retrospective assessment observed in this study need to be considered. Interestingly, fatigue was a positive predictor of accepting MS apps. Potentially, the current need to manage subjective fatigue may have increased the general openness to use innovative self-help tools. Therefore, it would be conceivable that the intention to use MS apps may exist regardless of fatigue, whereas fatigue may represent a barrier for actually using MS apps. In this respect, the role of fatigue should be explored in the context of an actual behavior with a sample with more clinically relevant cases of fatigue. Potentially, people suffering from severe fatigue might have not participated.

### Limitations

This study has different limitations. First, this observational study does not allow for causal conclusions because of the cross-sectional nature. Therefore, the results should be interpreted with caution. A next step would be to longitudinally assess the actual usage as predicted by acceptance. In line with prior research, we evaluated behavioral intention to use as a predictor of usage, but the direct translation to actual behavior is problematic (behavior-intention gap in technology use [83]). A next step for an observational study in cooperation with MS centers could be to include a follow-up assessment. Another option could be to conduct a randomized controlled trial to systematically assess the impact of acceptance interventions [84] on the actual uptake of MS apps in primary care (see the Implications section).

Second, the data were collected via a Web-based survey. Hence, disadvantages such as selection bias should be considered, as well as the high attrition rate of about one-third. This might have also contributed to low variance of fatigue severity among survey completers. Besides fatigue, cognitive difficulties could be another reason for noncompletion. However, most participants who dropped out did so after the first 3 demographic questions (year of birth, gender, and postal code). This indicates further reasons such as decline of interest or motivation.

Furthermore, the sample was quite small and not representative, although the gender ratio, the mean age, and duration of MS were comparable with the Bavarian data from the German MS registry [85]. However, this anonymous survey provided no option to verify such self-reported outcomes. We collected no data (such as medical reports) to confirm the diagnosis of MS. We only asked for the year of diagnosis, but not for the exact diagnosis or subtype of MS. We tried to only reach people with MS by using a selective recruitment strategy with the support by a German MS society.

Third, the narrowed scope on MS apps in Germany restricts the generalizability to other eHealth contexts relevant for MS-related self-help activities. Future studies should use an umbrella term or involve more eHealth and mHealth options. As prevalence rates of MS are highest in Northern European countries [2,3], we are confident that our findings can be applied to a broader MS population.

Fourth, the majority of the assessed constructs were scored using translated and modified scales, which might have compromised the reliability of the scale effort expectancy. In this study, however, we avoided using longer scales to reduce participant burden and to minimize the attrition rate. A next step would be to assess the validity of the transadapted UTAUT-related measure using a larger sample.

Fifth, the experience with MS apps was rather low. However, we assume that the proportion of participants in this self-selected Web-based sample who know about mHealth apps and have previously used them is higher than that in a primary care setting. The study was conducted in Germany where the mHealth or eHealth adoption in health care is at an early stage, but as panel surveys show, there is already public awareness of digital self-help options to some extent [86]. Furthermore, on average, this sample reported receiving an MS diagnosis a decade ago. Therefore, this and the connection to MS self-help organizations indicate openness to (online) self-help.

Finally, it should be also mentioned that the retrospective assessment of subjective fatigue is subject to methodological limitations. Furthermore, fatigue can be associated with cognitive deficits [87]. For a more accurate assessment of fatigue in a target group with different manifestations of MS, the use of longer scales combined with neurological tests would be worth considering.

### Implications

Although mobile phones belong to the everyday life of people with MS, their use in connection with MS seems relatively low [19]. In this Web-based study among smartphone users with MS, the vast majority (63.3%) reported no experience with MS apps. In contrast, the use of smartphones for MS-related purposes was higher. It can be assumed that the demand and use of eHealth or mHealth services vary over time. For example, a qualitative study by Colombo et al [88] indicated that MS patients find the internet useful for disease management purposes, but there can be a barrier at the beginning and later stages of MS to actively search information online because it is perceived as stressful. Consequently, MS apps can not only be a resource, but at the same time, such tools can also provide a medium for illness representations. Vaughan et al [89] showed that illness representations in people with MS reflect the medical knowledge about MS, in which the consequences of impending degeneration and lacking prospect of cure are salient. Such representations are linked with the concept of pathogenesis, and labels such as *MS apps* could underline this deficient perspective.

In contrast, the concept of salutogenesis [90] does not deny the challenges of MS, but it raises awareness on how to use one’s own resources to cope with them. In this sense, it can be assumed that a salutogenetic perspective could promote a greater motivation for health promotion, for instance, supported by apps. Possibly, the relatively high proportion of people in this sample who used nonspecific mHealth services rather than MS apps could indicate that they are avoiding stigmatization by the label *MS*. Hence, these considerations could be useful to improve the awareness of suitable apps and for the development of transdiagnostic apps for MS, with an emphasis on salutogenetic aspects. Another factor that should be considered is that there is not *one MS* but diverse manifestations that are related to different challenges and self-help preferences.

With performance expectancy being the strongest predictor of acceptance of MS apps, it can be suggested that the benefits of mHealth apps should be transparently communicated to potential users with MS [10]. Given the significant role of social influence in this study, it can be further recommended that such information should not just be provided via social media. In particular, a next step would be to rethink the integration of the social environment in the treatment of people with MS using eHealth and mHealth solutions.

In view of the limited experience with MS apps in this sample, before assessing the acceptance, more detailed information about digital self-management tools could be provided via acceptance-facilitating interventions (AFI). Short, video-based AFI have been shown to be effective in German primary care settings, for instance, in improving acceptance of digital interventions among patients with depression [84] and pain [91]. However, the results on the efficacy of AFI in Web-based study settings are inconsistent. For instance, a Web-based randomized controlled trial by Lin et al [92] showed no significant effects of a video-based AFI on the acceptance, adherence, and uptake rate of a mobile phone–based and Web-based intervention for chronic pain among people with long-term conditions. The (baseline) acceptance was, however, found to be higher than that observed the in target populations in primary care.

As there is a lack of such studies on the uptake of eHealth solutions for people with MS, it appears reasonable to develop AFIs with existing tools such as the commonly used *MS Kognition* in a routine care setting to assess the actual use predicted by acceptance. For this purpose, it is crucial to consider quality standards of apps to identify appropriate tools via app stores [93,94], especially with regard to disease management for long-term conditions [95]. Moreover, it appears likely that people with different forms of MS experience different challenges that need to be considered when assessing the adoption of MS apps and other self-help services. Hence, the MS form should be considered in future studies. It is also possible that the low adoption rate is also associated with the poor quality of many mHealth apps available via app stores. Hence, it is crucial to understand and systematically involve the perspectives of users in the quality improvement of mHealth apps.

Finally, there is a lack of suitable measures to determine the acceptance of eHealth solutions in specific populations and contexts. In line with other research [35,36,84,91,96], we thus chose to adapt the UTAUT framework to our target population and service of interest. It is, thus, important to validate such adapted UTAUT measures with large samples in upcoming studies. This could be a first step to achieve methodological consensus on the assessment of the acceptance and use of mHealth apps for the disease management of MS and allow for comparisons with other research. Moreover, the UTAUT framework is only one option to evaluate subjective views of people with MS on mHealth services. Attitudes may be more suitable for the assessment of early adoption of eHealth or mHealth [35]. In addition, other types of eHealth tools could be relevant for coping with MS in the target population, including psychological services. User perspectives could also be measured using validated measures such as the Attitudes towards Psychological Online Interventions [97]. Potentially, the evaluation of both acceptance and attitudes as well as related constructs could provide a more complete picture of the needs and preferences for different digital self-help tools among people with MS.

### Conclusions

Taken together, this study suggests that the intention to use MS apps is rather poor among the majority of participants without usage experience and that positive expectations about the helpfulness and social influence are important predictors for the acceptance of MS apps. Moreover, noteworthy is the use of MS-unspecific apps by almost half of the participants. This finding makes the investigation of the acceptance and use of MS-specific mHealth services in comparison with other self-management options appear a logical next step.

## Appendix

Multimedia Appendix 1 Questionnaire of the conceptual study model.

Multimedia Appendix 2 Additional descriptive data.

Multimedia Appendix 3 Additional regression analysis.

## Abbreviations

AFI: acceptance-facilitating interventions
CASMIN: Comparative Analysis of Social Mobility in Industrial Nations
CNS: central nervous system
C-SE: computer self-efficacy
DMSG: Deutsche Multiple Sklerose Gesellschaft
eHealth: electronic health
GCSE: general computer self-efficacy
G-eHEALS: German eHealth Literacy Scale
IU: Intolerance of Uncertainty
IUS: Intolerance of Uncertainty Scale
mHealth: mobile health
MS: multiple sclerosis
MS-SE: multiple sclerosis self-efficacy
NARCOMS: North American Research Committee on Multiple Sclerosis
SCT: social cognitive theory
TAM: technology acceptance model
TPB: Theory of Planned Behavior
UTAUT: Unified Theory of Acceptance and Use of Technology
