# Supplementary material for: Acceptance of Mobile Health Apps for Disease Management Among People With Multiple Sclerosis: Web-Based Survey Study
Source: JMIR Form Res. 2018 Dec 12;2(2):e11977. doi: 10.2196/11977 (PMC6334710; doi:10.2196/11977)
Supplement: Multimedia Appendix 1 [file formative_v2i2e11977_app1.pdf]

Multimedia Appendix 1

Questionnaire of the extended study model

Overview of scales and items of the conceptual study model: adapted and extended UTAUT-model

Table S1. Questionnaire for the assessment of core constructs of the conceptual study model.

**Scale**	**Performance Expectancy (PE)**	**UTAUT Predictor**
Source	Original: Hennemann et al. [1]	Adaptation in English (German)
PE1	A Web-based aftercare could improve my work-related well-being.	Using mobile MS apps could improve my general health-related well-being.<br>(Die Nutzung von MS-Apps könnte mein allgemeines gesundheitliches Wohlbefinden verbessern.)
PE2	A Web-based aftercare could help me with work-related stress	Using MS apps could help me to better deal with disease-related stress.<br>(Die Nutzung von mobilen MS-Apps könnte mir helfen, besser mit krankheitsbezogenem Stress umzugehen.)
PE3	A Web-based aftercare could help me to improve my personal health.	Using MS apps could help me to maintain physical mobility.<br>(Die Nutzung von MS-Apps könnte mir helfen, körperlich mobil zu bleiben.)
PE4	A Web-based aftercare could help me with my health problems.	Using MS apps could help me stay mentally fit.<br>(Die Nutzung von MS-Apps könnte mir helfen, geistig fit zu bleiben.)
PE5	Additional item (self-constructed)	Using MS apps could help me maintain social contacts.<br>(Die Nutzung von mobilen MS-Apps könnte mir helfen, soziale Kontakte zu pflegen.)
**Scale**	**Effort Expectancy (EE)**	**UTAUT Predictor**
Source	Original: Hennemann et al. [1], Venkatesh et al. [2]	Adaptation in English (German)
EE1	A Web-based aftercare would be easy to operate and comprehend.[1]<br>I would find the system easy to use.[2]	I suspect that using MS apps would be easy.<br>(Ich vermute, dass die Anwendung von MS-Apps leicht ist.)
EE2	I could arrange using a Web-based aftercare in my everyday life.[1]	I could easily integrate the use of MS apps into my everyday life.<br>(Ich könnte die Anwendung von MS-Apps leicht in meinen Alltag integrieren.)
**Scale**	**Social Influence (SI)**	**UTAUT Predictor**
Source	Original: Hennemann et al. [1]	Adaptation in English (German)
	(----)	The following people would approve it if I used MS apps:<br>(Folgende Personen würden es gut finden, wenn ich MS-Apps nutzen würde:)
SI1	People close to me would approve a Web-based aftercare.	Members of my family

		(Mitglieder meiner Familie)
SI2	My general practitioner would approve the use of a Web-based aftercare.	My general practitioner<br>(Mein mich hauptsächlich betreuender Arzt)
SI3	My friends would approve a Web-based aftercare.	My friends<br>(Meine Freunde)
**Scale**	**Facilitating Conditions (FC)**	**UTAUT Predictor**
Source	Original: Hennemann et al. [1], Venkatesh et al. [2]	Adaptation in English (German)
FC1	I have the necessary technical preconditions for using a Web-based aftercare.[1]<br>I have the resources necessary to use the system.[2]	I have the necessary technical preconditions to be able to use MS apps<br>(Ich verfüge über die nötigen technischen Voraussetzungen, um MS-Apps nutzen zu können.)
FC2	I possess the required technical knowledge to utilize a Web-based aftercare.[1]<br>I have the knowledge necessary to use the system.[2]	I have the required technical knowledge to utilize MS apps.<br>(Ich verfüge über das notwendige technische Wissen, um MS-Apps nutzen zu können.)
FC3	*Additional item (self-constructed)*	If I had problems using MS apps, I would know where to get help.<br>(Wenn ich Probleme bei der Anwendung von MS-Apps hätte, wüsste ich, wo ich mir Hilfe holen kann.)
**Scale**	**Behavioral intention to use MS apps (BI)**	**UTAUT Acceptance Criterion**
Source	Original: Hennemann et al. [1]' Venkatesh et al. [2]	Adaptation in English (German), based on the groups: User (1), Non-user (2), Previous user (3)
BI1	I would like to try a Web-based aftercare.[1]	I would like to try MS apps again (1, 3)<br>(Ich würde MS-Apps noch einmal ausprobieren. (1, 3))<br>I would like to try MS apps (2)<br>(Ich würde MS-Apps gern einmal ausprobieren. (2))
BI2	I would use a Web-based aftercare if offered to me.[1]	I would also use MS apps in the future. (1)<br>(Ich würde MS-Apps auch in der Zukunft nutzen. (1))<br>If I had the opportunity, I would use MS apps. (2)<br>(Wenn ich die Möglichkeit dazu hätte, würde ich MS-Apps nutzen. (2))<br>If I had the opportunity, I would use MS apps again. (3)<br>(Wenn ich die Möglichkeit dazu hätte, würde ich MS-Apps wieder nutzen. (3))
BI3	I intend to use the system in the next <n> months.[2]	I would not want to do without MS apps anymore. (1)<br>(Ich würde auf MS-Apps nicht mehr verzichten wollen. (1))<br>I intend to use MS apps in the next 4 weeks. (2)<br>(Ich beabsichtige, MS-Apps in den nächsten 4 Wochen zu nutzen. (2))<br>I intend to use MS apps again in the next 4 weeks. (3)<br>(Ich beabsichtige, MS-Apps wieder in den nächsten 4 Wochen zu nutzen. (3))
**Scale**	**Intolerance of Uncertainty (IU)**	**Extended UTAUT Predictor**

Source	Original: Buhr & Dugas [3]	Adaptation and translation in German
IU1	When it's time to act, uncertainty paralyses me.	When it's time to act, uncertainty associated with my disease rather paralyses me.<br><br>(Die mit meiner Erkrankung einhergehende Unsicherheit lähmt mich eher, als dass ich etwas dagegen unternehmen könnte.)
IU2	My mind can`t be relaxed if I don`t now what will happen tomorrow.	*Not adapted, only translated*<br><br>Es fällt mir schwer mich zu entspannen, wenn ich nicht weiß, was morgen passieren wird.
IU3	I can`t stand being taken by surprise.	*Not adapted, only translated*<br><br>Ich kann es nicht ertragen, wenn unvorhergesehene Dinge passieren.
IU4	Unlike me, others seem to know where they are going with their lives.	*Not adapted, only translated*<br><br>Im Gegensatz zu mir scheinen andere Leute zu wissen, in welche Richtung ihr Leben läuft.
**Scale**	**eHealth Literacy (eHL)**	**Extended UTAUT Predictor**
Source	Original: Soellner et al. [4]	Adaptation in German (replacement of health information with information about MS)
eHL1	Ich weiß, welche Quellen für Gesundheitsinformationen im Internet verfügbar sind.	Ich weiß, welche Quellen für Informationen zu MS im Internet verfügbar sind.
eHL2	Ich weiß, wo im Internet ich nützliche Gesundheitsinformationen finden kann.	Ich weiß, wo ich im Internet nützliche Informationen zu MS finden kann.
eHL3	Ich bin in der Lage, Informationen, die ich im Internet finde, kritisch zu bewerten.	Ich bin in der Lage, Informationen, die ich im Internet zu MS finde, kritisch zu bewerten.
eHL4	Ich kann im Internet zuverlässige von fragwürdigen Informationen unterscheiden.	Ich kann im Internet zuverlässige von fragwürdigen Informationen unterscheiden.
**Scale**	**MS Self-Efficacy (MS-SE)**	**Extended UTAUT Mediator**
Source	Original: Airlie et al. [5]	Adaptation in English (German)
MS-SE1	I feel in control of my life.	Despite my MS disease, I feel in control of my life.<br><br>(Trotz meiner MS-Erkrankung glaube ich, selbst über mein Leben bestimmen zu können.)
MS-SE2	The way my MS affects me in the future mostly depends on me.	*Not adapted, only translated*<br><br>(Es hängt zum großen Teil von mir selbst ab, wie die MS-Erkrankung mein Leben zukünftig beeinflussen wird.)
MS-SE3	Despite my difficulties, I still manage to cope with daily life.	Despite my difficulties, I still manage to cope with daily life with MS.<br><br>(Trotz der Herausforderungen mit MS schaffe ich es, meinen Alltag zu bewältigen.)
MS-SE4	I am confident I can overcome my difficulties.	I am confident I can overcome my daily difficulties with MS.<br><br>(Ich bin zuversichtlich, dass ich die täglichen Herausforderungen mit MS bewältigen kann.)
**Scale**	**Computer Self-Efficacy (C-SE)**	**Extended UTAUT Mediator**

Source	Original: Wang et al. [6]	Adaptation in English (German, replacement of computer with smartphone)
C-SE1	I believe I have the ability to install new software applications on a computer.	I believe that I have the ability to install new apps on my smartphone.<br>(Ich glaube, dass ich in der Lage bin, neue Apps auf meinem Smartphone zu installieren.)
C-SE2	I believe I have the ability to identify and correct common operational problems with a computer.	I believe I have the ability to correct technical problems with my smartphone.<br>(Ich glaube, dass ich in der Lage bin, technische Probleme mit meinem Smartphone zu korrigieren.)
C-SE3	I believe I have the ability to remove information from a computer that I no longer need.	I believe I have the ability to remove apps from my smartphone I no longer need.<br>(Ich glaube, dass ich in der Lage bin, Apps, die ich nicht mehr benötige, von meinem Smartphone zu löschen.)
**Scale**	**Fatigue (Fat)**	**Extended UTAUT Moderator**
Source	Original: D`Souza [7]	Adaptation and translation in German
	Because of my fatigue during the past 4 weeks …	*Not adapted, only translated*<br>Aufgrund von Fatigue hatte ich in den vergangenen 4 Wochen …(hatte ich…)
Fat1	I have been less alert.	Schwierigkeiten, aufmerksam zu sein.
Fat2	I have been limited in my ability to do things away from home.	Schwierigkeiten, etwas außerhalb meines Zuhauses zu tun.
Fat3	I have had trouble maintaining physical effort for long periods.	Schwierigkeiten, körperliche Anstrengung für einen längeren Zeitraum aufrechtzuerhalten.
Fat4	I have been less able to complete tasks that require physical effort.	Probleme, Aufgaben zu erfüllen, welche körperlicher Anstrengung bedürfen.
Fat5	I have had trouble concentrating.	Schwierigkeiten, mich zu konzentrieren.

References

1. Hennemann S, Beutel ME, Zwerenz R. Drivers and Barriers to Acceptance of Web-Based Aftercare of Patients in Inpatient Routine Care: A Cross-Sectional Survey. J Med Internet Res 2016;18(12):e337. PMID:28011445
2. Venkatesh, Morris, Davis. User Acceptance of Information Technology: Toward a Unified View. MIS Quarterly 2003;27(3):425. doi:10.2307/30036540
3. Buhr K, Dugas MJ. The Intolerance of Uncertainty Scale: Psychometric properties of the English version. Behav Res Ther 2002;40(8):931-945. PMID:12186356
4. Soellner R, Huber S, Reder M. The Concept of eHealth Literacy and Its Measurement. Journal of Media Psychology 2014;26(1):29-38. doi:10.1027/1864-1105/a000104
5. Airlie J, Baker GA, Smith SJ, Young CA. Measuring the impact of multiple sclerosis on psychosocial functioning: The development of a new self-efficacy scale. Clin Rehabil 2001;15(3):259-265. PMID:11386395
6. Wang D, Xu L, Chan HC. Understanding the continuance use of social network sites: A computer self-efficacy perspective. Behaviour & Information Technology 2014;34(2):204-216. doi:10.1080/0144929X.2014.952778
7. D'Souza E. Modified Fatigue Impact Scale - 5-item version (MFIS-5). Occup Med (Lond) 2016;66(3):256-257. PMID:27016749
