# Supplementary material for: Acceptance of Mobile Health Apps for Disease Management Among People With Multiple Sclerosis: Web-Based Survey Study
Source: JMIR Form Res. 2018 Dec 12;2(2):e11977. doi: 10.2196/11977 (PMC6334710; doi:10.2196/11977)
Supplement: Multimedia Appendix 2 [file formative_v2i2e11977_app2.pdf]

Multimedia Appendix 2

Additional descriptive data

Table S2. Sample characteristics (N=98)[a-h].

Variables		N = 98
Demographic sample characteristics		
**Age**	Mean (SD), range, years	47.03 (10.17), 22-67
	Women	45.11 (10.09), 22-67
	Men	51.0 (9.28), 26-66
	20-35 years, n (%)	16 (16.3)
	36-50 years, n (%)	43 (43.9)
	51-67 years, n (%)	39 (39.8)
**Gender**	Female, n (%)	66 (67.3)
	Male, n (%)	32 (32.7)
**Housing situation**	Living alone, n (%)	25 (25.5)
	Living with one person, n (%)	40 (40.8)
	Living with two or more persons, n (%)	33 (33.7)
**Secondary education**[a-d]	Certificate of secondary education[a], n (%)	8 (8.2)
	General certificate of secondary education[b], n (%)	27 (27.6)
	Advanced technical college entrance qualification[c], n (%)	19 (19.4)
	General qualification for university entrance[d], n (%)	44 (44.9)
**Vocational training and tertiary education**[e]	No professional qualification, n (%)	4 (4.1)
	Training qualification[e], n (%)	62 (63.3)
	Polytechnic or college degree, n (%)	9 (9.2)
	University degree, n (%)	23 (23.5)
Disease-related sample characteristics		
**Duration of MS**	M (SD), range (years)	13.92 (9.84), 1-45
	1-10 years, n (%)	43 (43.9)
	11-21 years, n (%)	37 (37.7)
	>21 years, n (%)	18 (18.4)

**Degree of disability**[f] (card for severely handicapped persons)	No physical disability, n (%)	11 (11.2)
	Disability of less than 50 percent, n (%)	27 (27.6)
	Disability of at least 50 percent, n (%)	58 (59.2)
	Missing, n (%)	2 (2.0)
**Experienced cognitive impairment**	No cognitive impairment, n (%)	51 (52.0)
	Yes, cognitive impairment, n (%)	44 (44.9)
	Missing, n (%)	3 (3.1)
**Diagnosed cognitive impairment**[g]	No formal diagnosis, n (%)	59 (60.2)
	Yes, diagnosed cognitive impairment, n (%)	28 (28.6)
	Not sure, n (%)	11 (11.2)
**Fatigue**[h]	M (SD)	3.31 (1.17)

[a]German “Hauptschulabschluss” as basic school qualification

[b]German secondary school level I certificate (“Mittlere Reife”)

[c]German “Fachhochschulreife”

[d]German "Allgemeine Hochschulreife” ("Abitur“ or A-Level)

[e]German dual training model

[f]Existence of a degree of disability according to the definition by the German rehabilitation law SGB IX (established by a medical board, self-reported item)

[g]Existence of a formal diagnosis of cognitive diagnosis, according to the International Classification of Diseases (ICD-10; diagnosed by a medical doctor, self-reported item)

[h]Fatigue was assessed using the 5-item Modified Fatigue Impact Scale (MFIS-5; D´Souza, 2016) on a five-point Likert-type scale ranging from 1 ("fully disagree") to 5 (“fully agree"). Higher scores indicate a higher level of fatigue (self-reported subjective impairment).

Additional descriptive analyses: non-specific mobile phone use

General use of smartphones/mobile phones involved text messages or emails (90.8%, 89/98), phone calls (77.6%, 77/98), internet access (71.4%, 70/98), scheduling (56.1%, 55/98), and music and games (33.7%, 33/98). Miscellaneous use (14.2%, 14/98) included, for instance, for social networks, navigation and photos (missing: 1.0%, 1/98). About half of the participants (52.0%, 51/98) did not use any apps.

MS-specific mobile phone use: utilization of specific MS apps

Table S3. Currently and previously used German MS apps (n=36).

App name (provider)	n=36
“MS Kognition” ([MS cognition], DMSG), n (%)	15 (41.7)
“KKNMS” app, not specified (KKNMS), n (%)	4 (11.1)

“MS und ich“ ([MS and me], Novartis AG), n (%)	1 (2.8)
“Aktiv mit MS“ ([active with MS], TEVA GmbH), n (%)	3 (8.3)
“MS-Tagebuch” ([MS diary], DMSG), n (%)	3 (8.3)
myBETAapp“ (BAYER AG), n (%)	2 (5.6)
“Gemeinsam stark“ ([strong together], MSlife, Biogen GmbH), n (%)	1 (2.8)
“Aubagio” app (Sanofi AG), n (%)	1 (2.8)
“Tecfidera” app (Biogen GmbH), n (%)	1 (2.8)
“MS und ich“ ([MS and me], Novartis AG), n (%)	1 (2.8)
“MS-TV“ (MSlife, Biogen GmbH), n (%)	1 (2.8)
“MSlife” app, not specified (MSlife, Biogen GmbH), n (%)	1 (2.8)
“MS block” (unknown provider), n (%)	1 (2.8)
“BeActive” (MSlife, Biogen GmbH), n (%)	1 (2.8)

Notes. Responses were provided by the participants as free text in a commentary field and summarized by the authors. Abbreviations: DMSG=Deutsche Multiple Sklerose Gesellschaft; KKNMS=Krankheitsbezogenes Kompetenznetz Multiple Sklerose (German for Disease-related competence net multiple sclerosis). All apps were provided and used in German (for Android and iOS). Multiple answers were possible within a commentary field. The provider of each app was added by the authors.

Table S4. Perceived benefits and challenges of MS apps (subsample of n=13): a qualitative synthesis of open answers.

	Subjective benefits (n=13)	Challenges (n=10)
**Performance expectancy – in relation to eHL**	▫ Improving cognitive performance<br>▫ Information and education about MS and treatment options, medication, barrier-free locations, health-related topics, cognition<br>▫ Information which apps are useful<br>▫ Maintaining social contacts	▫ Perceived overload through cognitive tests, demanding after short time<br>▫ Underperforming experience in the sense of little interesting
**Effort expectancy**	(----)	▫ Complicated/difficult login and logout, installation of apps only for users of a specific medication possible
**Perceived behavioral control (facilitating conditions)**	▫ Overview of the course of MS through documentation (cognition, memory, health)<br>▫ Ensure adherence to treatment (planning, documentation, reminder for medication, medical appointments)<br>▫ Quality control – good feeling by making sure the doctor is right	▫ Restricted export and import and synchronization options, so that there are restrictions on the process control

Ideas for improvements (n=7)

- Import and export of the generated data via cloud synchronization via cloud or similar
- Apps that test cognitive performance have no reference values (e.g., average of healthy / MS patients after x years of disease duration), which may be quite good for self-esteem reasons ;)
- Better overview of offers of help, grants etc.
- Even more different exercises to improve memory. Simple explanations to MS and medications
- Availability of apps for a wider range of mobile phones, expansion of offerings (e.g., event calendars, research findings)

Notes. Summary of answers to categories was based on open questions. All answers were translated to from German language. Out of N=98, n=13 participants used the voluntary option to answer the two open questions on subjective benefits and challenges, with 13 responses falling into the "benefits" category and 10 responses in the "challenges" category. Multiple answers per person were possible. The two open answers on subjective benefits and challenges were summarized by two researchers and mapped based on the UTAUT framework. The third open question on ideas for improvements was answered by 7 participants with written content. Of the 7 responses, 5 were valid in terms of concrete suggestions. The answers for the third question ideas for improvements were taken literally and translated to English.
