# Supplementary material for: Acceptance of Mobile Health Apps for Disease Management Among People With Multiple Sclerosis: Web-Based Survey Study
Source: JMIR Form Res. 2018 Dec 12;2(2):e11977. doi: 10.2196/11977 (PMC6334710; doi:10.2196/11977)
Supplement: Multimedia Appendix 3 [file formative_v2i2e11977_app3.pdf]

Multimedia Appendix 3

Additional regression analyses

Correlation analyses

Table S5. Correlation matrix for the variables in the adapted and extended UTAUT model for MS apps (N=98).

	BI	PE	EE	SI	FC	IU	eHL
BI	1.00	.736**	.336***	.707**	.265**	.267**	-.105
PE	-	1.00	.273**	.678**	.267**	.232*	-.047
EE	-	-	1.00	.421**	.516**	-.078	.170
SI	-	-	-	1.00	.268**	.298**	-.193
FC	-	-	-	-	1.00	-.194	.299**
IU	-	-	-	-	-	1.00	-.311**
eHL	-	-	-	-	-	-	1.00

Notes. Abbreviations*:* BI=behavioral intention to use MS apps (acceptance of MS apps); PE=performance expectancy; EE=effort expectancy; SI=social influence; FC=facilitating conditions; IU=intolerance of uncertainty; eHL=eHealth literacy. ***P* <.01; **P* <.05.

Regression analyses

As shown in Table S6, the simple regression analyses revealed that performance expectancy (beta=.74), effort expectancy (beta=.37), social influence (beta=.71), facilitating conditions (beta=.27) and IU (beta=.27) proved to be significant positive predictors for intentions to use MS apps (all *P*<.01). In contrast, eHealth literacy had no meaningful predictive contribution (beta=-.11, *P*=.303).

Table S6. Results of the simple regression analyses of the adapted and extended UTAUT-model for MS apps (N=98).

Predictors	R^2	*B*	beta	*SE*	*P*
Performance expectancy	.54	.99	.74	.09	<.001
Effort expectancy	.11	.55	.37	.16	.001
Social influence	.50	.91	.71	.09	<.001
Facilitating conditions	.07	.44	.27	.16	.008
Intolerance of uncertainty	.07	.35	.27	.13	.008
eHealth literacy	.01	-.20	-.11	.19	.303

Notes. Dependent variable: behavioral intention to use MS apps (help-seeking intention). *B*=unstandardized regression coefficient, beta=standardized regression coefficient, SE=standard error (for beta).

Influencing Variables: Mediator and Moderator Analyses

Mediator analyses

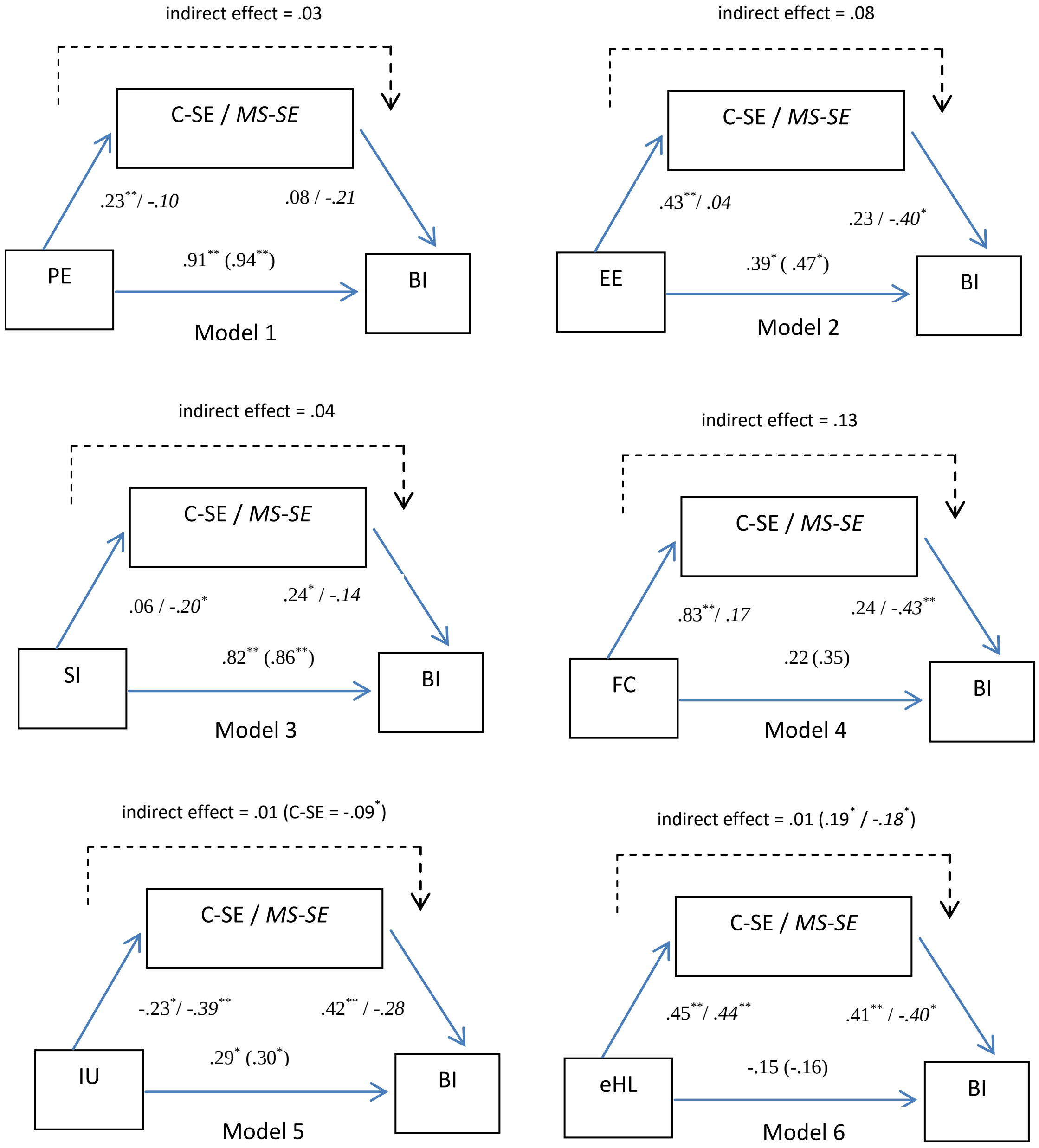

Figure S1. Results of the multiple mediation analyses for computer-self-efficacy and MS-related self-efficacy in the relationships between the predictors and behavioral intentions to use MS apps.

Notes. Behavioral intentions to use MS apps in terms of acceptance of MS apps. Both mediators were included simultaneously. Values before the slash = effects for computer self-efficacy (C-SE), italicized values behind the slash = effects for MS self-efficacy (MS-SE). Indirect effect refers to both mediators (C-SE, MS-SE). Effect of the predictor on the criterion: direct effect (in brackets = total effect). Abbreviations*:* BI=behavioral intentions to use MS apps; PE=performance expectancy; EE=effort expectancy; SI=social influence; FC=facilitating conditions; IU=intolerance of uncertainty; eHL=eHealth literacy. $^{**}P$ <.01; $^{*}P$ <.05.

The control variable age showed significant influence on C-SE in models 1, 3, 5, 6 (B=-.03, *P*<.001) and 2 (B=-.02, *P*<.02). In model 4 (FC), the effect remained marginal (B=-.01, *P*=.06). Gender as a control variable also proved to be a significant influence on C-SE in the models 1 (B=-.41, *P*=.04), 2 (B=-.46, *P*=.02), 3 (B=-.03, *P*<.001), 5 (B=-.45, *P*=.02) and model 6 (B=-.48, *P*<.01), meaning that younger and male participants had a stronger expression of C-SE.

Moderator analyses

Table S7. Mean values of effort expectancy and behavioral intention to use MS apps depending on the fatigue levels.

Effort expectancy	Fatigue	Behavioral intention to use MS apps
M = 3.0	*M* - 1 *SD* = 2.14	*M* = 2.6
M = 3.8	*M* = 3.31	*M* = 3.1
M = 4.6	M + 1 *SD* = 4.48	*M* = 4.0

Notes. Scales ranging from 1 (min) to 5 (max). Simple-slope analyses using SPSS for low (Mean-1 SD) and high (Mean+1 SD) levels of the proposed moderator fatigue. With average and high levels of fatigue, a greater positive influence of effort expectancy (perceived ease of use) on the criterion (behavioral intention to use MS apps in terms of acceptance) was found. The covariate duration of the MS disease showed a significant influence in the models 2 (EE-BI), 4, and 6 (eHL-BI) on the criterion intention to use MS apps (B=-.03, *P*<.05). All other control variables showed no significant influence.

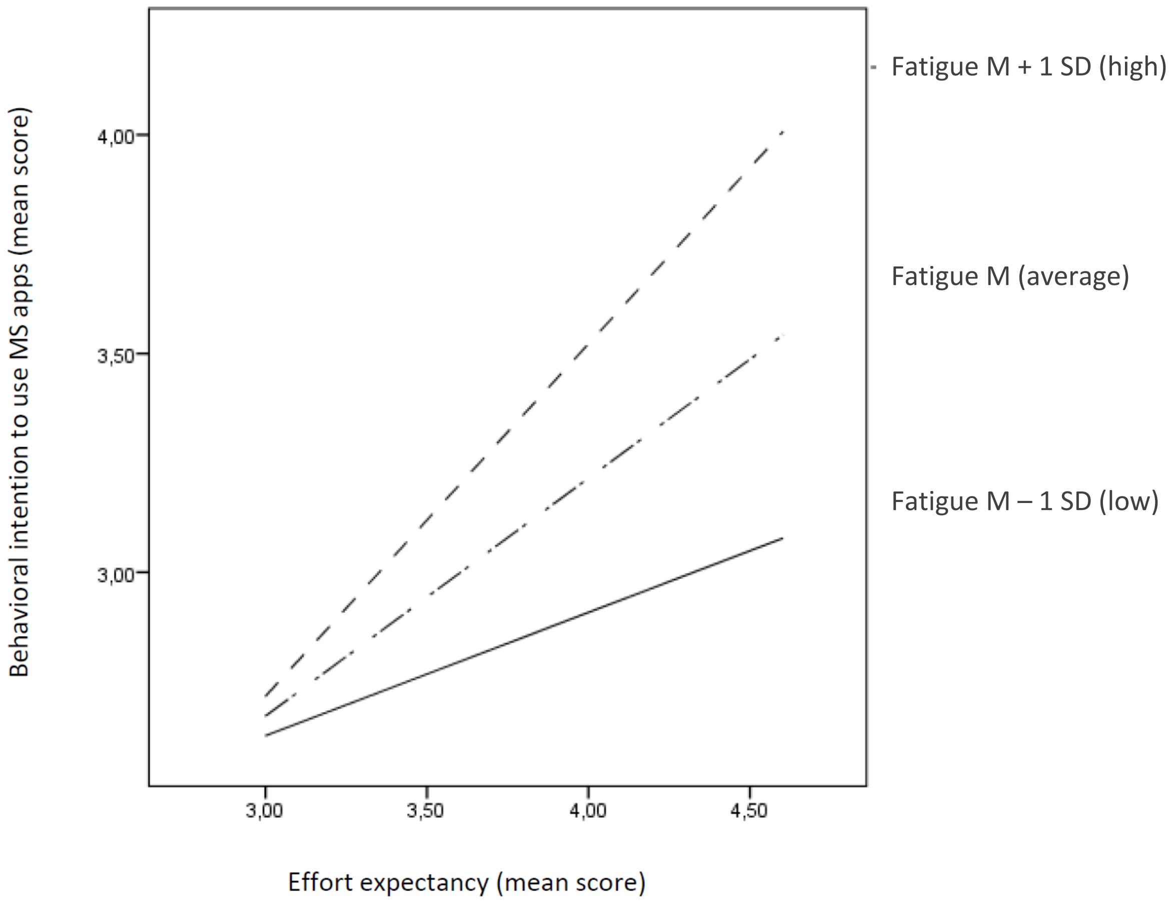

Figure S2. Marginal interaction of effort expectancy x fatigue in behavioral intention to use MS apps (*P*=.078). A significant moderation effect on the relationship between effort expectancy (perceived ease of use) and behavioral intentions to use MS apps (acceptance) was only shown in case of average and high levels of the moderator fatigue.
